# [*SP*-4-2]-(Aceto­nitrile-κ*N*)chlorido­[2-(4,6-di­phenyl­pyridin-2-yl)phenyl-κ^2^ *C* ^1^,*N*]platinum(II). Corrigendum

**DOI:** 10.1107/S2414314620001558

**Published:** 2020-02-28

**Authors:** Chengjian Fang, Zhao Wang, Zhongjian Cong, Shengli Li, Fei Li

**Affiliations:** a Department of Chemistry, Anhui University, Hefei, Anhui 230039, People’s Republic of China

**Keywords:** crystal structure, platinum(II) complex, *C*,*N*-chelating ligand, corrigendum

## Abstract

The article by Fang *et al.* [*IUCrData* (2019), **4**, x191207] is withdrawn.

## Abstract 2

Withdrawal of *IUCrData* (2019), **4**, x191207.

We wish to withdraw the article by Fang *et al.* (2019 ▸). The article was based on structural data previously reported by Zhu (2014 ▸).
